# Grain density and its impact on grain filling characteristic of rice: mechanistic testing of the concept in genetically related cultivars

**DOI:** 10.1038/s41598-018-22256-2

**Published:** 2018-03-07

**Authors:** Kaushik Das, Binay B. Panda, Birendra P. Shaw, Satya R. Das, Sushanta K. Dash, Ekamber Kariali, Pravat K. Mohapatra

**Affiliations:** 1 Environmental Biotechnology Laboratory, Institute of Life Sciences, Bhubaneswar, 751023 India; 2 Department of Plant Breeding and Genetics, Orissa University of Agriculture and Technology, Bhubaneswar, 751003 India; 3 National Rice Research Institute, Cuttack, 753006 India; 4 School of Life Sciences, Sambalpur University, Jyoti vihar, Sambalpur, 768019 India

## Abstract

Physiological factors controlling assimilate partitioning was compared in relation to panicle architecture of lax- (Upahar) and compact-panicle (Mahalaxmi) rice cultivars. Grain number and ethylene production at anthesis are low, but filling rate is high in the former compared to high grain number and ethylene production and poor filling trait of the latter. Similar to Mahalaxmi, its progenitors Pankaj and Mahshuri, had attributes of high grain number and grain density, but grain filling was higher and ethylene evolution was lower. Disturbed genetic coherence owing to imbalance of gene groups brought in the cross combinations of Mahshuri and Pankaj could be responsible for high ethylene production leading to semi sterility of Mahalaxmi as the hormone slackened endosperm starch bio-synthesis enzyme activities. Mahalaxmi inherited grain compactness trait of its progenitors, but not the physiological attribute for reduced ethylene production, which impacted grain filling. Upahar, the progeny genotype of Mahalaxmi and IR62 cross, inherited the dominant allele for low ethylene production and good grain filling traits from the high yielding IR62. In conclusion grain filling in compact-panicle rice becomes poor subject to expression of recessive allele for high ethylene production, but the allele is amenable for suppression by corresponding dominant allele in a genetic breeding.

## Introduction

Panicle architecture in rice specifying numbers of primary and secondary branches, spikelets per panicle, length of the branches and primary rachis and density of spikelets, determines sink based yield potential of rice^1–4^. Because of the agronomic importance, panicle architecture is a major target for rice domestication in different agro-climatic conditions. It is suggested that rice antiquity embraced transition of a spread type panicle into compact type in course of domestication; a compact panicle cultivar bearing more seeds in the panicle had advantage for high grain yield^5^. The change over of panicle architecture has involved modification of several morphological and physiological characters. In sharp contrast to the heterogeneous architecture, the typical trait of spreading type panicle, spikelets distribution of compact panicle is homogeneous with each spikelet borne in close proximity of the other. In the process, metabolic dominance of the apical spikelets is compromised^6^, and reduction of sink strength widens the gradient in assimilate partitioning between individual grains of panicle. Several authors have compared development of grains located at different positions of rice panicle in cultivars contrasting for grain compactness and reported increase in margin of variation of quality and average weight uniformity commensurate with increase of grain density^7–9^. Most of the poorly developed grains are located on the secondary branches, especially on the basal part of the panicle^10^. The metabolic dominance of spikelets for assimilate partitioning is dependent on the expression of genes for the endosperm starch synthesizing enzymes^11^ and the genes express poorly in inferior spikelets because of high ethylene synthesis and enhanced transduction of its signal^12^.

In pursuit of achieving higher grain yield potential rice breeders have increased panicle spikelet number to more than 250 in the new plant type rice by introduction of germplasm derived from tropical *japonica* rice^13^, but the desired objective of harnessing high yield could not be achieved due to poor grain filling. The compact panicle *japonica* cultivars have been introduced into different parts of the rice growing nations of the World, especially China^9^. They bear advantage of 20–25% higher yield compared to the IR8 parented semi-dwarf rice. A similar endeavour in *indica* cultivars has not been successful owing to lack of studies in finding the relationship between panicle architecture and grain filling. Grain yield has seldom exceeded 10 t ha^−1^; far below the predicted maximum approximating 15 t ha^−1^ ^14,15^. Hence, suggestions have been made to scale down the panicle spikelet number from 250 to 125 for improving grain filling in the revised version of new plant type rice^13^. This proposition, implemented in the absence of scrutiny of genotypic diversity in grain filling among the compact-panicle cultivars will significantly undermine scope for achieving high rice yield. Several alleles for good grain filling exist in *indica* cultivars; they should be investigated and incorporated to extra-heavy panicle types of rice^16^. In our previous studies, high ethylene production at anthesis has been considered a potent factor detrimental for spikelet filling in compact-panicle cultivar Mahalaxmi^12^, but the genealogy in expression of this undesirable allele and its distribution among different cultivars of similar panicle type are not known hitherto. The objective in the present experiment is to assess the inheritance pattern in expression of the allele for ethylene evolution at anthesis in semi-sterile compact-panicle cultivar Mahalaxmi and its suppression in the progeny type cultivar Upahar, resulting from a cross between Mahalaxmi and IR62.

## Results

### Morphological features

Compared to Mahalaxmi, Upahar had larger plant size, produced more tillers, greater panicle breadth and higher grain weight and grain filling percentage (Table 1). In Mahalaxmi panicle size was equal to that of Upahar, but it had larger number of grains. Grain density was high and most of the inferior type grains were borne on secondary branches of the basal part of panicle. Compared to superior, the inferior grains had very low dry mass. High grain density attribute (small inter-grain space) of Mahalaxmi was comparable to that of its parents Pankaj and Mahshuri; more particularly the latter. However, the yield attributes of parents, more specifically, Mahshuri did not match perfectly with its progeny genotype Mahalaxmi. Both Pankaj and Mahshuri had large number of secondary branches and spikelets, but grain filling in them was not poor like that of Mahalaxmi. Additionally, the wide gradient in grain weight between superior apical and inferior basal spikelets of Mahalaxmi was found in neither Mahshuri nor Pankaj. Although tiller number did not differ between the parental and filial generations, plant height was significantly lower in the latter compared to the former. Cross breeding of Mahalaxmi with IR62 restored some useful grain filling traits in Upahar. Grain laxity and filling increased at the cost of reduction of grain number. IR62 had marginal difference in weight of apical and basal grains and this attribute was reflected in the filial generation Upahar. Data on hulled and de-hulled grain size of the five cultivars did not match entirely with the gradient in grain weight between apical and basal spikelets of the cultivars, although size of the former was larger than the latter in all cultivars (Table 2). Upahar inherited the low panicle grain number and density attributes from IR62. Grain number was more than 200 in cultivars Mahalaxmi, Mahshuri and Pankaj, significantly higher than Upahar and IR62. Grain density was very high in both Mahshuri and Mahalaxmi, compared to low density of the other three cultivars. These two cultivars had more numbers of secondary branches, which contributed to increase of grain number and density. The percentage of un-filled grains was low in the low grain density cultivars, Upahar, IR62 and Pankaj. In the compact-panicle cultivars Mahalaxmi and Mahshuri, percentage of un-filled grains was high in the former, but not in the latter (Table 1).

**Table 1:** Morphological features of rice cultivars. ±Values indicate SD of 3 replicates. Means followed by common letters within a column show no significant difference in Duncan’s multiple range test (DMRT).

Cultivars	Plant height (cm)	Productive tiller no.	Panicle wt. (g)	Panicle length (cm)	Panicle grain no.	Apical 1000 grain wt. (g)	Basal 1000 grain wt. (g)	No. of Primary branches	Inter Primary branch space (cm)	No. of Secondary branches	Unfilled grain (%)	Inter-grain space (cm)	Grain density (Grain no.cm^−1^ panicle)
Mahalaxmi	114.26 ± 1.7d	11.00 ± 1.0c	3.84 ± 0.1b	25.76 ± 0.4d	264.00 ± 20.5b	21.73 ± 1.5b	12.88 ± 1.1b	12.00 ± 1.0b	2.24 ± 0.1b	45.00 ± 2.6b	46.06 ± 1.3a	0.10 ± 0.0c	9.24 ± 0.3b
Upahar	134.66 ± 3.6b	18.33 ± 1.5a	3.45 ± 0.1c	26.90 ± 0.9c	162.00 ± 5.2e	26.27 ± 1.0a	24.47 ± 1.2a	11.33 ± 1.1b	2.16 ± 0.1b	23.33 ± 2.3c	17.51 ± 2.9b	0.17 ± 0.0a	5.97 ± 0.5d
IR62	124.83 ± 4.0c	14.66 ± 2.3b	3.63 ± 0.0b	31.10 ± 0.9a	195.33 ± 4.1d	27.34 ± 0.4a	26.00 ± 0.6a	10.66 ± 0.5b	2.92 ± 0.2a	23.00 ± 2.0c	5.09 ± 1.9c	0.15 ± 0.0a	6.28 ± 0.1d
Mahshuri	143.66 ± 2.1a	11.33 ± 1.5b	5.49 ± 0.1a	27.26 ± 0.2c	315.66 ± 11.9a	18.23 ± 0.5c	12.92 ± 0.6b	15.66 ± 1.1a	1.74 ± 0.1c	62.66 ± 3.7a	8.43 ± 1.7c	0.08 ± 0.0d	11.57 ± 0.4a
Pankaj	130.76 ± 0.6b	13.33 ± 2.5b	5.32 ± 0.1a	29.10 ± 0.7b	216.33 ± 5.6c	26.62 ± 0.5a	24.90 ± 0.8a	10.66 ± 0.5b	2.73 ± 0.1a	41.00 ± 2.6b	8.49 ± 2.1c	0.13 ± 0.0b	7.42 ± 0.3c
5% LSD	4.96	3.38	0.28	1.18	20.59	1.73	1.72	1.42	0.29	4.99	3.82	0.02	0.69

**Table 2:** The effects of hull removal on grain size of rice cultivars. ±Values indicate SD of 3 replicates. Means followed by common letters within a column show no significant difference in Duncan’s multiple range test (DMRT).

Cultivars	Grain Position	Grains with hull			Grains de-hulled		
		Grain Length (mm.)	Grain Width (mm.)	Grain Thickness (mm.)	Grain Length (mm.)	Grain Width (mm.)	Grain Thickness (mm.)
Mahalaxmi	Apical	7.615 ± 0.14c	2.855 ± 0.09c	1.810 ± 0.08c	5.375 ± 0.07d	2.438 ± 0.09c	1.558 ± 0.05c
	Basal	7.570 ± 0.12c	2.876 ± 0.16c	1.420 ± 0.14e	5.348 ± 0.18d	2.043 ± 0.13e	1.366 ± 0.07d
Upahar	Apical	8.358 ± 0.34b	2.956 ± 0.05c	1.755 ± 0.05c	5.900 ± 0.17b	2.330 ± 0.06d	1.546 ± 0.05c
	Basal	8.088 ± 0.30b	2.798 ± 0.08d	1.830 ± 0.04c	5.981 ± 0.09b	2.320 ± 0.06d	1.573 ± 0.05c
IR62	Apical	8.308 ± 0.22b	3.351 ± 0.08a	2.070 ± 0.06a	5.776 ± 0.13c	2.746 ± 0.07a	1.820 ± 0.07a
	Basal	7.795 ± 0.22c	3.091 ± 0.15b	2.116 ± 0.07a	5.348 ± 0.18d	2.580 ± 0.09b	1.815 ± 0.05a
Mahshuri	Apical	7.611 ± 0.10c	2.481 ± 0.12e	1.703 ± 0.03d	5.365 ± 0.05d	2.105 ± 0.11e	1.521 ± 0.04c
	Basal	7.386 ± 0.17d	2.433 ± 0.08e	1.695 ± 0.04d	5.086 ± 0.09e	1.983 ± 0.06 f	1.505 ± 0.09c
Pankaj	Apical	8.765 ± 0.23a	2.800 ± 0.10d	1.976 ± 0.04b	6.293 ± 0.18a	2.358 ± 0.04c	1.731 ± 0.04b
	Basal	8.816 ± 0.31a	2.905 ± 0.07c	1.945 ± 0.02b	5.86 ± 0.14b	2.370 ± 0.05c	1.746 ± 0.05a
5% LSD		0.27	0.12	0.08	0.16	0.10	0.07

### Grain weight

**Figure 1:**
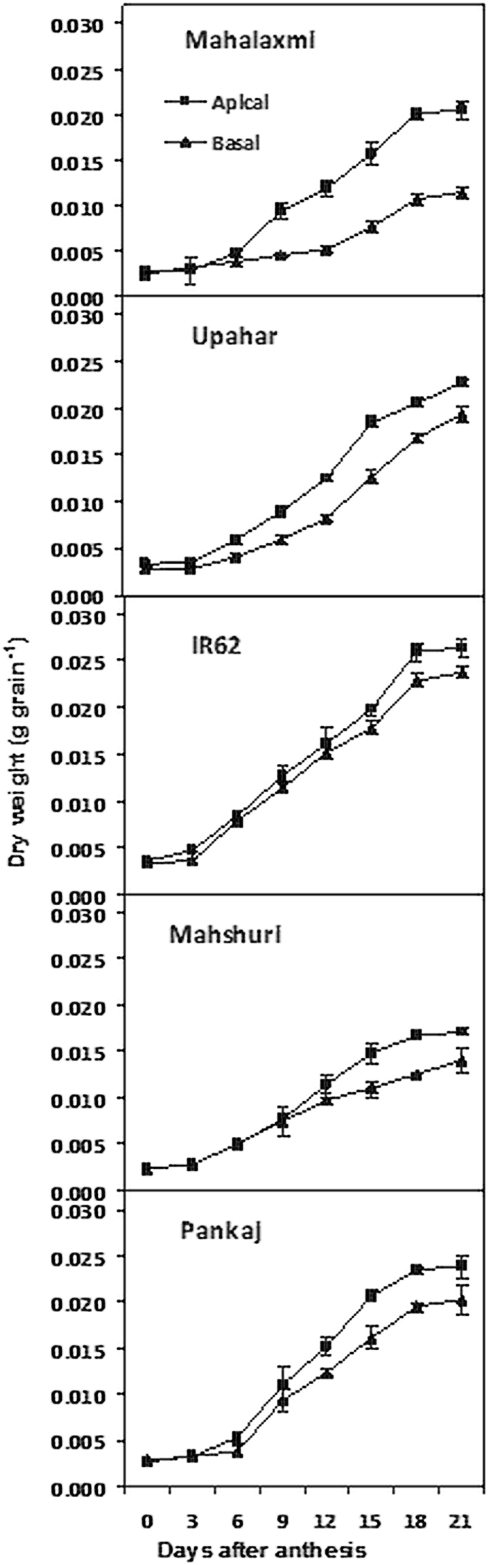
Dry weight of the apical and basal grains of panicle of the rice cultivars Mahalaxmi, Upahar, IR-62, Mahshuri and Pankaj during post anthesis period. Vertical bars represent ±SD of 3 replicates. The variance between apical and basal grains and on different days after anthesis in each cultivar was significant at *P* ≤ 0.01 tested by balanced ANOVA in CROPSTAT software.

Grain dry weight increased temporally in both apical and basal grains in an S-shaped curvilinear pattern during the post anthesis period in all the five cultivars examined (Fig. 1). The rate of grain growth was poorer in the basal compared to the apical spikelet. Both apical and basal grains of the panicle had larger dry mass and size in the lax-panicle cultivars, Pankaj, Upahar and IR62 in comparison to the compact-panicle cultivars Mahalaxmi and Mahshuri (Tables 1 and 2). Among the cultivars, the gradient in grain weight between apical and basal spikelets at maturity was highest in Mahalaxmi and it was followed by Mahshuri. In comparison, the other three cultivars did not exhibit similar difference in weight of the two types of spikelets during the whole period of observation. Gradient in grain size between the two types of spikelets of the cultivars did not always exhibit pattern similar to that of the differences in grain weight. Average grain weight of Mahshuri was quite low compared to the other four cultivars. Difference in dry mass was corroborated by observations taken for grain fresh weight of the cultivars (data not presented).

### Carbohydrate concentration of grains

**Figure 2:**
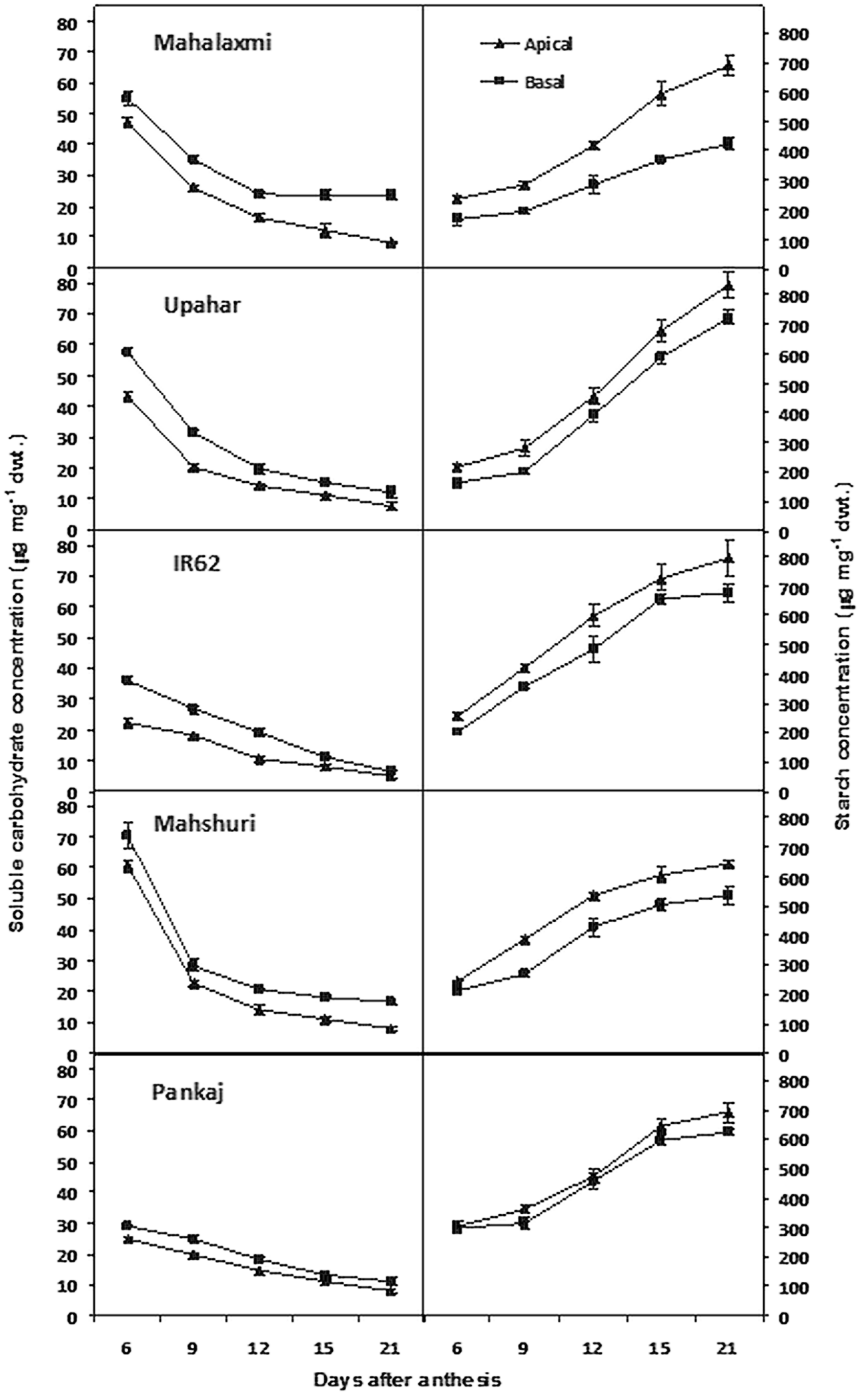
Soluble carbohydrates (left) and starch (right) concentration of apical and basal grains of panicle of rice cultivars Mahalaxmi, Upahar, IR-62, Mahshuri and Pankaj during post anthesis period. Vertical bars represent ±SD of 3 replicates. The variance between apical and basal grains and on different days after anthesis in each cultivar was significant at *P* ≤ 0.05 tested by balanced ANOVA in CROPSTAT software.

The concentration of soluble carbohydrates of grains was high during the first part of grain filling period in all the five cultivars (Fig. 2). It declined progressively with passage of time and reached low value at the grain hardening stage 21 DAA (days after anthesis). The concentration was higher in basal in comparison to the apical spikelets. Compared to other cultivars, the basal spikelet of Mahalaxmi accumulated more soluble carbohydrates and the gradient between concentration of apical and basal spikelets was highest in this cultivar. In this cultivar, the concentration did not decline temporally in the basal as much as that of the apical spikelet exhibiting a significant difference on 21 DAA. This trend was almost identical in its parent Mahshuri, but not in the other parent Pankaj that exhibited trend of decline in concentration similar to the lax-panicle cultivars Upahar and IR62.

In contrast to soluble carbohydrates, starch concentration of grains increased continuously with the progress of time in all cultivars. Grain starch concentration of the lax-panicle cultivars Upahar and IR62 was high and it was closely followed by Pankaj. In comparison, grains of both Mahalaxmi and Mahshuri garnered lower starch concentration. The grain starch concentration as well as temporal gain in concentration was lower in the basal spikelet compared to the apical spikelet. Among the cultivars, the difference in starch concentration of apical and basal spikelets during grain development was highest in Mahalaxmi and it was followed by Mahshuri.

### Activity of starch synthesizing enzymes of grains

**Figure 3:**
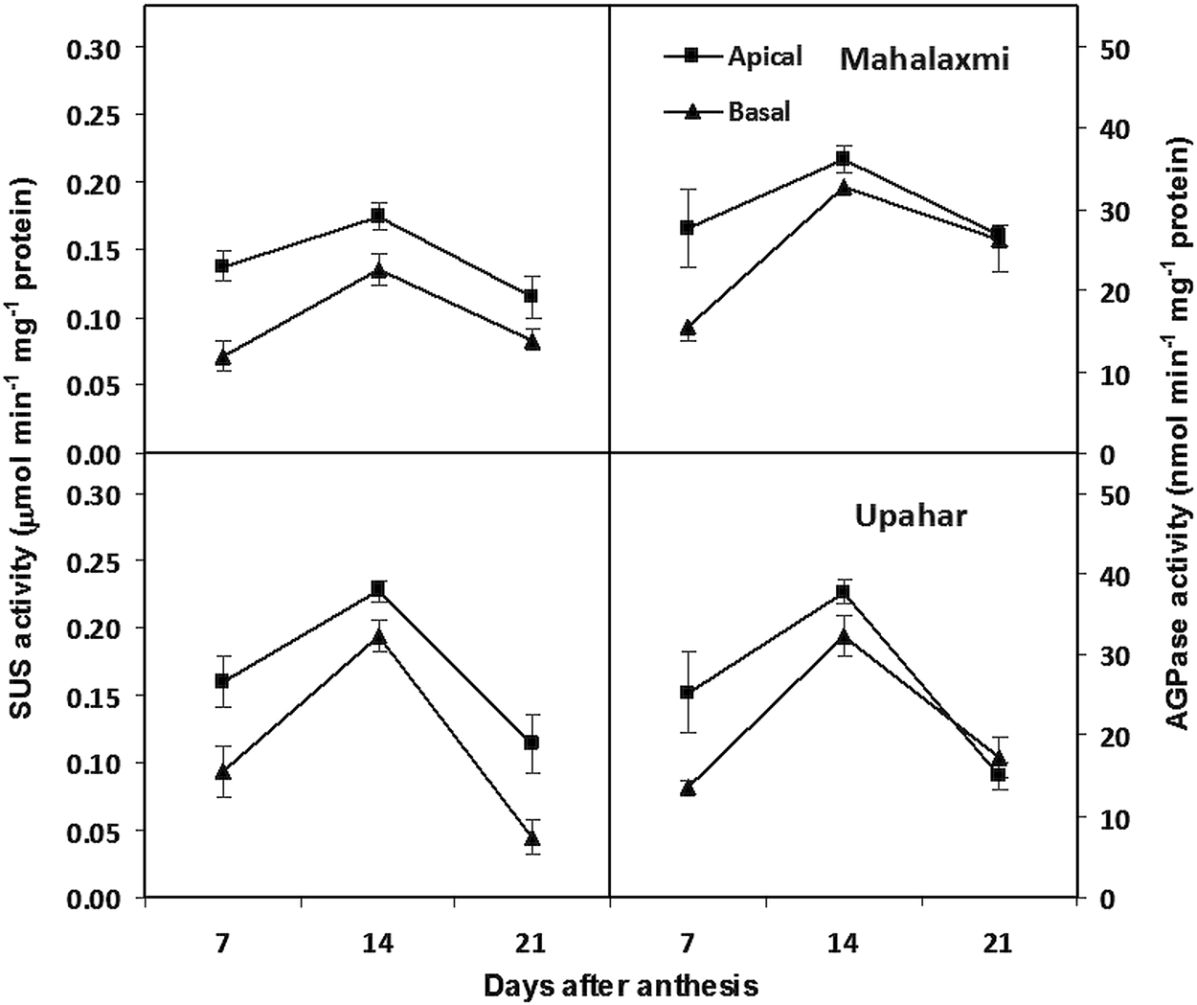
Sucrose synthase (left) and ADPGlucose pyrophosphorylase (right) activities of apical and basal grains of panicle of rice cultivars Mahalaxmi (upper case) and Upahar (lower case) during post anthesis period. Vertical bars represent ±SD of 3 replicates. The variance between apical and basal grains and on different days after anthesis in each cultivar was significant at *P* ≤ 0.01 tested by balanced ANOVA in CROPSTAT software.

Activity of sucrose synthase and ADPGlucose pyrophosphorylase enzymes in the developing grains was measured on three different occasions during the post anthesis period in cultivars Mahalaxmi and Upahar (Fig. 3). Enzyme activity was low at 7 DAA, it increased to a peak level at 14 DAA and declined thereafter briskly to an all time low at 21 DAA in both the cultivars. Enzyme activity was lower in the basal compared to apical spikelet. The difference in enzyme activity was quite discernible at 7 DAA and it was maintained at 14 DAA. But at 21 DAA, difference in activity was evident for sucrose synthase, but not for AGPase. At the peak level on 21 DAA, activities of both the enzymes were greater in the spikelets of Upahar in comparison to Mahalaxmi.

### Ethylene concentration of the spikelets

**Figure 4:**
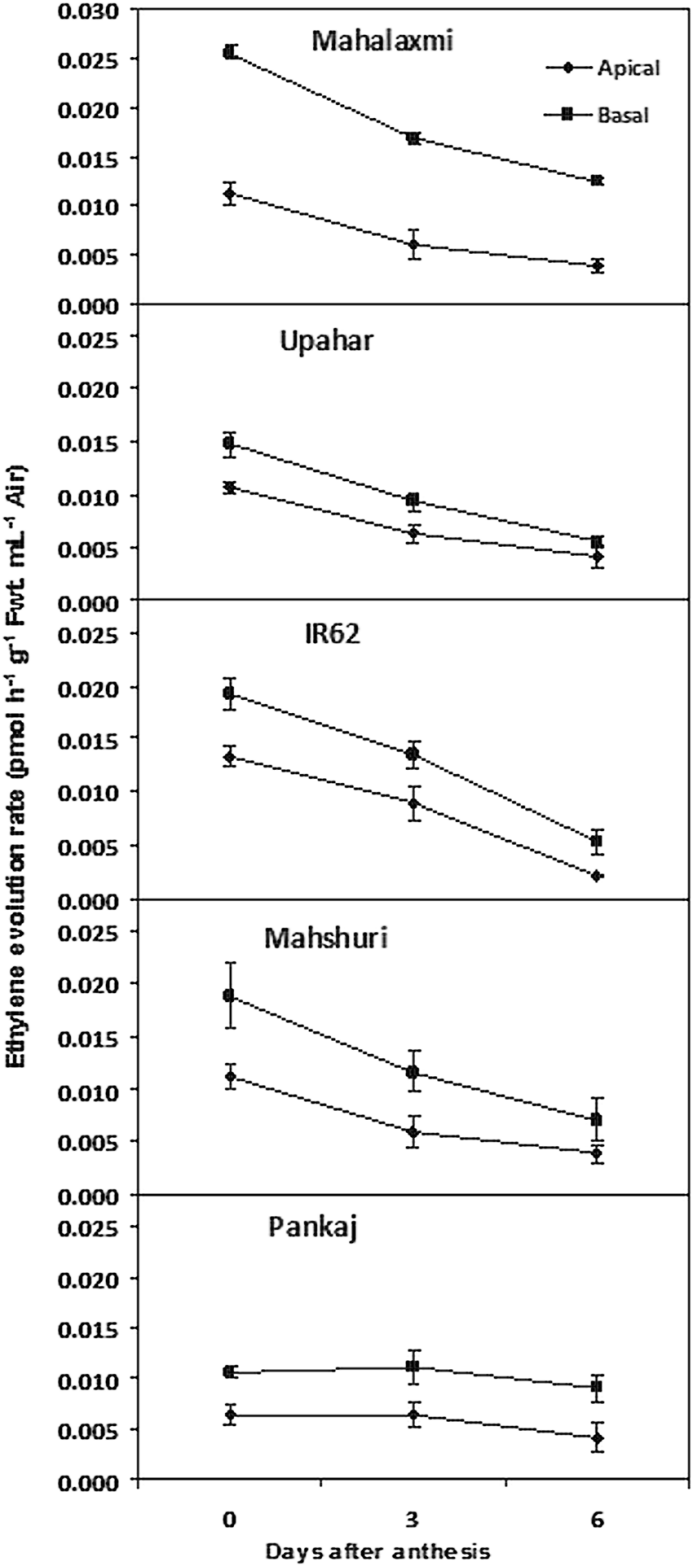
Ethylene concentration of apical and basal spikelets of panicle of rice cultivars Mahalaxmi, Upahar, IR-62, Mahshuri and Pankaj during post anthesis period. Vertical bars represent ±SD of 3 replicates. The variance between apical and basal spikelets in each cultivar was significant at *P* ≤ 0.05 tested by balanced ANOVA in CROPSTAT software.

Ethylene evolution rate of spikelets was high at anthesis in all the five cultivars and it declined progressively with the passage of time in the first six days after anthesis (Fig. 4). The basal spikelets produced more ethylene compared to the apical spikelets. Cultivar wise, ethylene production in basal spikelet was highest in Mahalaxmi. The concentration of ethylene in apical spikelet did not vary as much as that of basal spikelet in Mahalaxmi, Upahar, IR 62 and Mahshuri. In comparison, ethylene production was very low in both apical and basal spikelets of cultivar Pankaj. The gradient in ethylene production between apical and basal spikelets during the early post-anthesis period was wider in Mahalaxmi and Mahshuri compared to the other three lax-panicle cultivars.

## Discussion

Panda *et al*.^8^ observed that increased compactness of panicle allows space for accommodation of a larger number of spikelets, but it does not provide any advantage for improvement of grain yield potential. Grain filling is slackened because of high ethylene production and over-expression of genes encoding ethylene receptors and signal transducers^12^. The concept proposed holds good in the present experiment when comparisons of grain filling and ethylene evolution were made between lax-panicle cultivar Upahar with compact-panicle cultivar Mahalaxmi (Fig. 4). The latter possessed a large number of inferior type spikelets mostly on secondary branches (Table 1) and grain filling in them was low because of poor starch synthesis. Poor sink activity for starch synthesis resulted in accumulation of soluble assimilates in the inferior type spikelets (Fig. 2 and ref.^10^). This was corroborated by low activity of starch synthesizing enzymes (Fig. 3 and ref.^11^). On account of this physiological/biochemical bottle neck, the inferior spikelets amassed low dry mass that widened the gradient in weight between apical and basal grains (Table 1 and Fig. 1). In the light of these supportive evidences we can propose that ethylene evolution at anthesis is detrimental for panicle grain filling. The expression of this trait should be suppressed for improvement in yield potential of rice through genetic interventions or application of ethylene inhibitors^17,18^.

However, a closer examination of grain filling and physiological attributes of the parental genotypes Pankaj, Mahshuri and IR62 in comparison with their progeny genotypes Upahar and Mahalaxmi has provided an easy solution for poor grain filling attribute of the latter (Mahalaxmi) and resolve it with the identification of a hormonal factor, like ethylene with greater conviction. Both the parents of Mahalaxmi, namely Pankaj and Mahshuri contributed to its high grain number and density (Table 1); where contribution of the former was relatively lower than the latter. But the inheritance of grain number was dissociated from any possible identical lineage in grain filling. Grain filling was good in both the parents of Mahalaxmi and they produced less ethylene (Fig. 4). Sink efficiency for grain filling was reflected on greater activity of AGPase and SUS enzymes leading to higher starch synthesis in the developing grains and the consequential decline in concentration of soluble assimilates (Figs 2 and 3).We have reported earlier that genetic expression and activity of endosperm starch synthesizing enzymes were poorer in compact-panicle cultivars than the lax-panicle cultivars^11^. While the present results corroborate the earlier observations, there can be some explanations for poor spikelet filling of Mahalaxmi. As one of its parents Mahshuri is an *indica*-temperate *japonica* hybrid, therefore, in such cross combinations F1 was found to be highly fertile; but it segregates in F2 and subsequent generations to remain semi-sterile. It was assumed that operation of “duplicate fertility” gene, whose double recessive combination leads to pollen sterility^19,20^, and resultant reduced grain filling. Siddiq and Swaminathan^21,22^, suggested that the differentiation of *indica* and *japonica* species proceeded through a series of systematic mutations affecting distinguished characteristics brought together in a cluster probably under the influence of disruptive selection. They suggested that the disturbed genetic coherence rather than major chromosomal differences may be responsible for semi-sterility and skewed segregation ratios observed in the *indica/japonica* hybrid progenies. Some Japanese workers suggested that the sterility in the inter-subspecific hybrid is due to the absence of a few Mendelian sterility neutralizing (wide compatible) genes^23,24^.

While disturbed genetic coherence owing to imbalance of gene groups brought in the cross combinations of Mahshuri and Pankaj may be responsible for semi sterility of Mahalaxmi, our study has revealed a possible masking of the attribute. The cross between Mahalaxmi and the semi-dwarf high yield potential rice cultivar IR62 has resulted in progeny genotype Upahar, in which dominance of grain filling has surfaced at the cost of reduction in spikelet number and heterogeneous distribution of spikelets in the panicle^6^. Ethylene production was low in IR62 (Fig. 4) and this attribute has dominated over high ethylene production trait of Mahalaxmi. In other words, Mahalaxmi might have a double recessive allele for expression of high ethylene production. Additionally, Mahshuri is a versatile cultivar, where grain filling is higher and ethylene production is lower than Mahalaxmi, in spite of the fact that the grains are closely spaced in the panicle. Thus, closer packing improving homogeneity of grain development^8^ has come at the price of increased diversity of individual grain weight, because of the wide gap in weight between apical and basal grains (Table 1). Because grain size is a genotypic trait, it did not differ as much as that of grain weight between apical and basal spikelets (Table 2). The divergence of individual grain weight of panicle observed for Mahshuri has been almost similar to the other compact-panicle cultivar Mahalaxmi. Mohapatra *et al*.^17^ discovered the adverse effect of ethylene on grain filling in the inferior spikelets of rice panicle for the first time and suggested manipulation of its effects for improvement of grain filling through application of exogenous chemical inhibitors. This discovery has been corroborated by several recent reports^12,25,26^. In this context, the present study has brought out a novelty by identifying a genetic solution of the problem, wherein grain occurrence can be improved when ethylene production is minimised. However, genesis of ethylene may not be the sole factor controlling grain growth, when spikelets are distributed in close proximity of each other in a homogenous pattern. Interplay of positive and negative growth regulators maintaining hormonal balance might have been altered with variation in intrinsic capacity of cytokinin synthesis in the genotypes. Developing seeds are a rich source of cytokinin^27^. The role of cytokinin in grain filling, counter balancing adverse ethylene action, has been proposed in our earlier studies^28,29^ and that of the Chinese researchers^30,31^. In addition we also proposed ethylene as being a second messenger for IAA regulated spikelet dominance in rice panicle leading to growth inhibition of basal spikelets^12^. However, impact of grain density in rice panicle on cytokinin synthesis, although crucial for counter balancing ethylene action and thereby increasing grain occurrence, have not been studied.

Zhu *et al*.^5^ reported that lax-panicle architecture of wild rice is controlled by a dominant gene *OsLG1* and a single nucleotide polymorphism-6 in the cis-regulatory region 11 kb upstream of the translation start site can fine tune target gene expression leading to development of compact-panicle architecture in domesticated rice. Similarly, Adriani *et al*.^4^ found presence of QTL *qTSN4* responsible for increased total branch length, branching frequency and spikelet number per panicle; *qTSN4* effects on spikelet number depend on plants’ internal resources in particular the pre-floral accumulation of assimilates. Japanese researchers^32^ identified gene *SPIKE* from a tropical japonica landrace with potential of increasing rice yield of *indica* rice by 13–36% by increasing both source and sink sizes, in particular the spikelet number of panicle. Hasida *et al*.^33^ reported that a near isogenic line of rice carrying chromosome segment of *OsSPS1* of indica rice Kasalath in the genetic background of Koshihikari produces greater number of spikelets per panicle; *OsSPS1* encoding sucrose synthase activity of leaf could promote greater distribution of assimilates to panicle and increase spikelet number. Ectopic expression of an E-class gene of rice *OsMADS1*, specifying identities of lemma and palea, induced abnormal brassinosteroid and gibberellic acid activity that decreased number branches and spikelets per panicle^34^. However, seldom have we known the mechanism of gene/QTL expression controlling panicle size or spikelet number let alone grain yield through regulations in carbon partitioning or hormonal balance. In this context, our study is the first of its kind to find existence of a recessive allele, expression of which induces high ethylene production of spikelets to the detriment of grain filling in rice panicle and its dominance suppressing the undesirable effects. Further, we noticed that irrespective genetic variation, expression of the allele leading to the undesirable effects on grain occurrence is strongly linked with positional disadvantage of spikelets on panicle (Table 1; Figs 1 and 4). In our previous studies, treatments given to rice plant, such as external application of growth regulators^29^ and reduction of panicle size by partial excision of primary branches^35^ ameliorated the undesirable effects presumably by altering hormonal balance in favour of growth promotion. Hence, dominance of the recessive allele might have taken place through a possible up-regulation of genes controlling cytokinin biosynthesis. With technology amenable for genetic modifications and theoretical calculation permitting an upward revision of yield potential in rice^15^, scope exists for an ideal architecture of extra-large panicle embodying a balance between spikelet number and density for optimum grain filling. This revision of panicle architecture would be possible by modifying the inflorescence into a simple and indeterminate type with placement of spikelets more on primary than secondary branches^36^. In addition, compact panicle genotypes like Mahshuri should be studied empirically through biochemical and biotechnological interventions investigating contribution of alleles regulating both ethylene and cytokinin production during grain development in rice.

## Conclusion

Disturbed genetic coherence owing to imbalance of gene groups brought in the cross combinations of rice genotypes Mahshuri and Pankaj could be responsible for high ethylene production and semi sterility incident up on low sink activity for grain starch synthesis in the large and compact panicle progeny genotype Mahalaxmi. The adverse effects of high ethylene production on grain filling could be circumvented by reducing spikelet number and changing spikelet distribution into lax orientation in cultivar Upahar by cross breeding Mahalaxmi with semidwarf high yielding cultivar IR62. In general grain filling in compact-panicle rice becomes poor subject to expression of recessive allele for high ethylene production, but the allele is amenable for suppression by corresponding dominant allele in genetic breeding.

## Materials and Methods

### Genotypes and experimental site

The experimental material used in the present study included five rice cultivars namely Mahalaxmi, Upahar, IR62, Mahshuri and Pankaj differing with respect to panicle architecture and grain density. Mahalaxmi was derived from a cross between Pankaj and Mahshuri, while Upahar was a progeny genotype of Mahalaxmi and IR62. The seed materials were obtained from Department of Plant Breeding and Genetics, Orissa University of Agriculture and Technology, Bhubaneswar, India. The details of these genotypes are given in Table 3. The experiment was conducted under normal cultural conditions in the field of School of Life Sciences, Sambalpur University, India during the wet season in the year 2013. The experimental design was a randomized complete block design (RCBD) in three replications with a plot size of 6.0 (3 m × 2 m) square meters, row to row distance of 20 cm and plant to plant spacing of 15 cm. A check experiment similar in design was conducted at National Rice Research Institute, Cuttack for reference. Fertilizer dose of 100 N, 50 P_2_O_5_ and 50 K_2_O Kg per hectare was applied as per the scheduled management practices. The recommended crop management practices were followed including need based plant protection measures to raise a normal crop. The experiments were conducted with permission of authority of the institutions.

**Table 3:** Genealogy and phenotypic characteristics of rice cultivars Mahalaxmi, Upahar, IR62, Mahshuri and Pankaj.

Genealogy	General features
Mahalaxmi (Pankaj/Mahshuri)	· Semi-dwarf plant stature (95–100 cm)
	· Late maturity duration (150–155 days)
	· Profuse tillering habit
	· Intermediate panicle length (24 cm)
	· Heavy panicle weight type
	· High spikelet number per panicle
	· Spikelet fertility percentage 65–66%,
	· Straw coloured hull with medium bold grains
	· Photosensitive
	· Suitable for rainfed and irrigated low lands
	· Developed at Orissa University of Agriculture and Technology, Bhubaneswar.
Upahar (Mahalaxmi/IR 62)	· Intermediate plant stature (125 cm)
	· Late maturity duration (155–160 days)
	· Profuse tillering habit
	· Long panicle (27 cm)
	· Heavy panicle weight type
	· Moderate spikelet number per panicle
	· Spikelet fertility percentage 87–90%,
	· Straw coloured hull with short bold grains
	· Photosensitive
	· Suitable for both shallow and semi deep water lowlands
	· Developed at Orissa University of Agriculture and Technology, Bhubaneswar.
IR 62 (PTB 33/IR30 //IR 36)	· Semi-dwarf plant stature (98 cm)
	· Mid-early maturity duration (115–120 days)
	· Profuse tillering habit
	· Intermediate panicle length
	· Moderate spikelet number per panicle
	· Spikelet fertility percentage 85–90%,
	· Dark grey colour hull with medium slender grains
	· Photo-insensitive
	· Broad spectrum of resistance to major insect pests and diseases
	· Tolerant to salinity, iron toxicity and phosphorus deficiency
	· Suitable for rainfed and irrigated medium lands
	· Developed at IRRI, Philippines.
Pankaj (Peta/Tongkai Rotan)	· Intermediate plant stature (130 cm)
	· Mid-late maturity duration (140–145 days)
	· Profuse tillering habit
	· Intermediate panicle length
	· Moderate spikelet number per panicle with improved fertility
	· Straw coloured hull with medium bold grains
	· Weakly photosensitive
	· Broad spectrum of resistance to major insect pests and diseases
	· Tolerant to salinity and boron toxicity
	· Suitable for rainfed and irrigated low lands
	· Developed at IRRI, Philippines.
Mahshuri (Taichung 65/ Mayangebos 80/2)	· Tall plant stature (140 cm)
	· Mid-late maturity duration (145–150 days)
	· Moderate tillering habit
	· Intermediate panicle length
	· Moderate spikelet number per panicle with improved fertility
	· Spikelet fertility percentage more than 85%
	· Red coloured hull with medium slender grains
	· Weakly photosensitive
	· Suitable for medium and low lands of coastal areas during wet season
	· Developed at Malaysia from *indica japonica* cross.

### Sampling

On the day of first anthesis, the panicle on main shoot of the plants was tagged. This day was referred to as 0 DAA (days after anthesis) for the spikelets borne on the branches located in the apical region of panicle (apical spikelets). The spikelets borne on the branches in basal region of the panicle reached anthesis after 4–5 days and corresponded to 0 DAA for those spikelets (basal spikelets). The apical and basal spikelets were collected at 0, 3 and 6 DAA separately for ethylene analysis. Spikelet samples were also collected from 6 DAA at three day intervals till 21 DAA for assay of soluble carbohydrates and starch and at 7, 14 and 21 DAA for assays of the starch synthesizing enzymes. The collected samples for enzyme assay were placed separately in 15-mL Falcon tubes and were immediately frozen in liquid nitrogen at the sampling site. The frozen samples were stored at −80 °C until analysis. Facilities available at Institute of Life Science, Bhubaneswar, India were also used for the biochemical analyses with permission of the authorities.

### Grain phenotype investigation

Matured apical and basal grains were selected from each cultivar for the measurements of grain traits like grain length (GL), grain width (GW) and grain thickness (GT). Each individual grain as well as the de-husked endosperm was measured using an Indosaw Mitutoyo Absolute for these traits at National Rice Research Institute, Cuttack, India. The mean value of each trait was regarded as the phenotypic value, which was determined by six randomly selected full matured grains of each cultivar.

### Assay of soluble carbohydrates and starch

After recording the dry weight of kernels of apical and basal spikelets, the materials were boiled in 80% aqueous methanol for 15 min in a water bath. Then methanolic extract of the boiled material was transferred to a volumetric flask. The residue after methanolic extraction was boiled again with 50% aqueous methanol and both extracts were pooled together. The volume was made up to the mark with distilled water. Aliquots of the solution were taken for the estimation of soluble sugars by the addition of phenol sulphuric acid reagent by colorimetric method with OD taken at 490 nm using glucose as the standard^37^. The residue after methanolic extraction was dried and then boiled with 3% HCl for 3 hr in a water bath. After the acid hydrolysis of starch, the glucose released was measured in aliquots of diluted extracts according to phenol-sulphuric acid method^37^ and the value of glucose was converted to starch by multiplying with a factor of 0.9.

### Ethylene Assay

The apical and basal spikelets were transferred separately into a 5 mL glass tube containing 1 mL of distilled water. Then the tubes were incubated in darkness for 20 minutes to allow dissipation of the ethylene induced by cutting or wounding. The tubes were sealed thereafter by airtight rubber caps and incubated in darkness for two hours at room temperature. After incubation, 1 mL headspace gas was drawn from the individual tubes into a gas tight syringe and injected into the Gas Chromatograph (Model No. 6890, Hewlett-Packard Company, Palo Alto, CA, USA), equipped with flame ionization detector (FID) and micro-capillary column (length 30 m and internal diameter 0.53 mm) packed with cross-linked methyl siloxane for estimation of ethylene^38^. Nitrogen was used as the carrier gas whereas hydrogen and air were used for the flame. The instrument was calibrated with pure ethylene prior to the injection of gaseous samples into the Chromatograph. Ethylene concentration was expressed in pmole/hr/g.fwt/mL air.

### Enzyme assay

For assays of the starch synthesizing enzymes, 200 mg kernels of apical and basal rice spikelets each were ground in liquid nitrogen with a mortar and pestle. The ground sample was then homogenized in 1 mL of extraction buffer, which consisted of 50 mM HEPES-NaOH (pH 7.5), 2 mM MgCl_2_, 1 mM EDTA, 2.5 mm DTT, 12.5% (v/v) glycerol and protease inhibitor cocktail. The homogenate was centrifuged twice at 15,000 *g* for 15 min. The supernatant was stored in −80 °C and used subsequently as the crude enzyme extract.

### Sucrose synthase (SUS)

50 μL of enzyme extract was added to a micro test tube containing 25 μL 40 mM uridine 5′-diphosphate glucose (UDPG) and 50 μL 40 mM fructose, vortexed and the sample incubated at 30 °C for 15 min. After incubation, the mixture was neutralized by adding 175 μL 1 N NaOH. At the same time, 50 μL of sample extract was added to a second micro test tube containing 25 μL 40 mM UDPG, 50 μL 40 mM fructose and 175 μL 1 N NaOH. The reaction in both test tubes was stopped thereafter by boiling the sample for 10 min. Samples were cooled down and 250 μL resorcinol (0.1% w/v) in 95% ethanol and 750 μL concentrated HCl were added. Samples were floated in a water bath at 80 °C for 8 min along with a set of standards. The standards were prepared for 0, 100, 200, 300, 400 and 500 nmol sucrose. Before putting the standards in the water bath at 80 °C, 250 μL resorcinol and 750 μL concentrated HCl were added. Absorbance was measured at 520 nm in the incubated samples (30 °C for 15 min) and also for non-incubated samples. Sucrose synthase activity was determined from the difference in absorbance according to^39^.

### ADPglucosepyrophosphorylase (AGPase)

The assay was conducted at 30 °C in 100 mM HEPES NaOH buffer (pH 7.4) containing 3 mM 3-phosphoglycerate, 1.2 mM ADP Glucose, 3 mM sodium pyrophosphate, 5 mM MgCl_2_, 4 mM DTT, and 50 µl of crude enzyme extract in a reaction mixture volume of 250 µL. The reaction was allowed to continue for 20 minutes. The enzymes were inactivated thereafter by placing the mixture in a boiling water bath for 2 min. After addition of 350 µL of distilled water, the mixture was subjected to centrifugation at 15,000 *g* at 2 °C for 10 min. The supernatant (500 µL) was mixed with 0.15 mg NADP^+^. The enzymatic activity was recorded as the increase in absorbance at 340 nm after the addition of 1 µL each of phosphoglucomutase (0.4 units; Roche Diagnostics) and G6P dehydrogenase (0.5 units; Type XV, Sigma) with reference to Mohapatra *et al*.^29^.

### Data availability statement

All data generated or analysed during this study are included in this published article.
